# Related factors of quality of life of type 2 diabetes patients: a systematic review and meta-analysis

**DOI:** 10.1186/s12955-018-1021-9

**Published:** 2018-09-19

**Authors:** Xiyue Jing, Jiageng Chen, Yanan Dong, Duolan Han, Haozuo Zhao, Xuying Wang, Fei Gao, Changping Li, Zhuang Cui, Yuanyuan Liu, Jun Ma

**Affiliations:** Department of Health Statistics, College of Public Health, Tianjin Medical University, No. 22 Qixiangtai Road, Heping District, Tianjin, 300070 People’s Republic of China

**Keywords:** Type 2 diabetes, Quality of life, Related factors, Meta-analysis

## Abstract

**Background:**

Diabetes is a chronic disease, and it could affect both health and quality of life (QOL). A lot of studies have reported some predictors of QOL of type 2 diabetes patients. While their results were not completely consistent. So the aim of our study was finding out the related factors (including characteristics related to the disease, life styles and mental health factors) of QOL of type 2 diabetes patients.

**Methods:**

We searched Cochrane library, EmBase, PubMed and CNKI databases for published studies that evaluated the related factors of QOL of type 2 diabetes patients by using a proper statistic method and had effect sizes (*OR* or *β*) and 95% confidence intervals from January 1st 2000 to May 31st 2016. Any study types were acceptable, and we excluded the reviews, letters, editorials and pooled analyses. The data were analyzed using STATA software (Version 12.0; Stata Corporation). Effect sizes and 95% confidence intervals were calculated to evaluate the relationship between these factors and QOL.

**Results:**

Eighteen studies were included into our systematic review and meta-analysis, totaling 57,109 type 2 diabetes patients. Do more physical exercises (The pooled ORs ranged from 0.635 to 0.825 for different scales, less than 1.00), glucose check more frequently [pooled OR (95%CI): 0.175 (0.041, 0.756)] were associated with a better QOL. Presence of complications (The pooled ORs ranged from 1.462 to 3.038 for different scales, more than 1.00), presence of hypertension [pooled OR (95%CI): 1.389 (1.173, 1644)], longer duration of diabetes [pooled OR (95%CI): 1.865 (1.088, 3.197)], diet with more red meat [pooled OR (95%CI): 2.085 (1.063, 4.089)] and depression (The pooled ORs ranged from 3.003 to 11.473 for different scales, higher than 1.00) were associated with a worse QOL.

**Conclusion:**

The results of this study show that physical exercise, glucose check frequently, complications, hypertension, duration of diabetes, diet with more red meat, and depression were associated with the QOL of type 2 diabetes patients.

## Background

Diabetes is a chronic disease, and it could cause many serious short-term and long-term consequences [1] that affect both health and quality of life (QOL) [2]. The total number of diabetes patients worldwide may rise to about 370 million in 2030 from about 170 million in 2000 [3]. According to the World Health Organization (WHO), Type 2 diabetes patients accounts for 90% of all diabetes worldwide [4]. The morbidity of Type 2 diabetes mellitus also have been increasing over past decades [5].

QOL refers to a person’s individual perception of physical, emotional, and social status [6, 7]. Type 2 diabetes patients have great pressure to treat themselves, and they have lower QOL than those healthy persons [8, 9]. For chronic diabetes patients, a complete cure cannot be achieved [10]. Clinical measures can provide a good estimate of disease control, but the ultimate aim of diabetes care is preventing the patient’s QOL to get worse [10]. Understanding the predictors and identifying risk factors of QOL is important and these factors may then be targeted for prevention [6].

There are many different scales that could measure the QOL, such as EuroQol 5D (EQ-5D), Audit of Diabetes Depentent Quality of Life (ADDQoL), Diabetes-Specific Quality of Life (DSQL), Short Form-Series (SF-36, SF-8, SF-12, and so on), and so on. EQ-5D has five dimensions (Modility, Self-Care, Usual Activities, Pain/Discomfort and Anxiety/Depression) and each dimension has three levels [1]. The ADDQoL scale is composed of two overview items and 18 life domains [11]. The DSQL scale is a specific instrument for diabetes patients and is used to measure the QOL of Chinese diabetes patients. It includes 27 items and four domains: Physical Function, Psychology/Mind, Social Relation, and Influence of Treatment [12]. SF-36 consists of 36 questions that stand for 8 fields of life. Four fields stand for the Physical Health (PH): Physical Functioning (PF); Role limitations due to Physical health problems (RP); Bodily Pain (BP); General Health perceptions (GH). Another four fields stand for the Mental Health (MH): Vitality (VT); Social Functioning (SF); Role limitations due to Emotional problems (RE); and Emotional State (ES) [13].

Recently, many studies [1, 2, 5, 11–24] have reported some related factors of QOL of type 2 diabetes patients. While authors of these studies used different scales which were mentioned above. Take the factor “complication” for an example, Liu et al. [19] and Xie et al. [20] used the DSQL scale, while Aldona et al. [13] and Zu et al. [20] used the SF series scales to evaluate the QOL of type 2 diabetes patients. Meanwhile, for a same factor measured by a same scale, they even had different opinions about whether it was QOL’s related factor or not. Take the factor “Diet control” for an example, Zu et al. [20] showed the results that patients who controlled their diet would have a worse QOL than those who did not control diet. While Aldona et al. [13] showed that the factor “Diet control” was not associated with the QOL of type 2 diabetes patients.

For the reasons mentioned above (preventing the patient’s QOL to get worse is the ultimate aim of diabetes care while the opinions about related factors of QOL were not unitive by the previous researches), and considering the fact that many demographic characteristics could not be modified. The aim of our study was that after searching for studies which reported the related factors (including characteristics related to the disease, life styles and mental health factors) of QOL of type 2 diabetes patients across the internet databases, we pooled their results together via the systematic review and meta-analysis method, to understand the related factors of QOL of type 2 diabetes patients.

## Methods

### Search strategy and selection criteria

We have searched Cochrane library, EmBase, PubMed and CNKI (China National Knowledge Infrastructure) databases for published studies that evaluated the related factors of type 2 diabetes patients’ QOL from January 1st 2000 to May 31st 2016 (Because the WHO published the definition and diagnose criteria in 1999 [25], so we search the publications since 2000). The following search terms MeSH Terms and Text Word were used: “Quality of life”, “Type 2 diabetes” and “Factors”.

The following inclusion criteria were contained: (1) the related factors of type 2 diabetes patients’ QOL had been analyzed, and there were effect sizes and 95% confidence intervals (95% CI); (2) the minimum sample size was 30; (3) the language was limited in English and Chinese only; (4) the type of the publication was limited in “article”.

The exclusion criteria were as follows: (1) did not use a proper statistic method or lack effect sizes and 95% confidence intervals (95% CI), such as OR and β; (2) sample size was less than 30; (3) reviews, letters, editorials and pooled analyses; (4) did the investigation before the year 2000.

Two investigators did the study selection. They screened the titles, abstracts and full articles independently. Any disagreements about exclusion or inclusion of a study were resolved by a discussion with other two authors. And the quality of these articles were evaluated by a checklist with 11 items which was recommended by AHRQ (Agency for Healthcare Research and Quality). If an item was answered “NO” or “UNCLEAR”, it would be scored “0”; if it was answered ‘YES’, then it was scored “1”. The summary score of the 11 items would be the article’s quality score, which was assessed as follows: 0–3 = low quality; 4–7 = moderate quality and 8–11 = high quality. The articles with moderate quality or high quality would be included into this analysis [26].

**Fig. 1:**
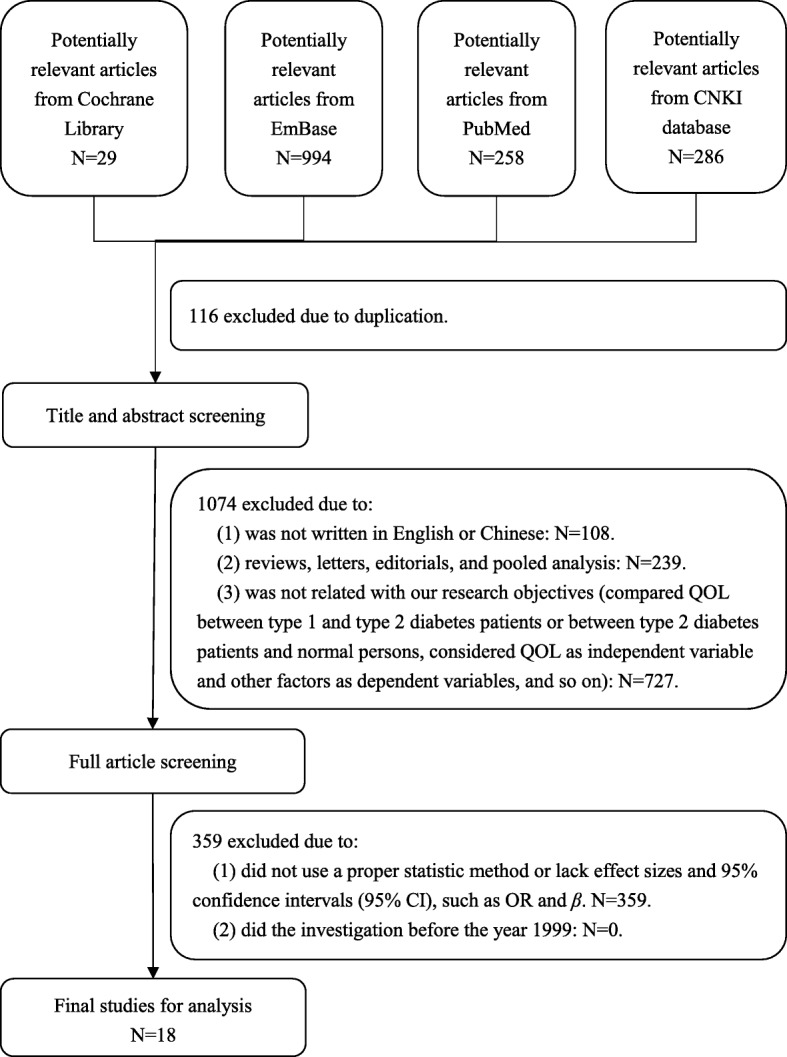
Flowchart showing the selection of studies for meta-analysis

After the search, 18 articles were entered into our analysis (Fig. 1).

### Data analysis

STATA software (Version 12.0; Stata Corporation) was used for data analyzing. *OR*s (for logistic regressions) or *β*s (for linear regression) with a 95% *CI* were calculated to evaluate the related factors of QOL of type 2 diabetes patients. *I*^*2*^ was calculated to assess the heterogeneity among our selected studies. A fixed-effects model was used if heterogeneity did not exist (*I*^*2*^ ≤ 50%). Otherwise, a random-effects model was employed (*I*^*2*^ > 50%). In addition, Egger’s test was used to analyze the publication bias in our selected studies. *P* value less than 0.05 was considered statistically significant.

## Result

### Study characteristics

As is shown in Table 1, all of the 18 included studies were questionnaire surveys (cross-sectional studies), with a total sample size of 57,109. The following different scales were used to research QOL of type 2 diabetes patients: EQ-5D, SF series (SF-36, SF-8, and SF-12), DMQLS, DSQL, ADDQoL. Several recent studies have documented the use of SF-36 and SF-12 are similar [27–31]. Similarly, the SF-8 uses one question to stand for each of the 8 SF-36 domains, and they have the same metric on single item scales and summary measures [32].

**Table 1:** Characteristics of the studies included in this meta-analysis

Study	Country	Study design	Sample size	Scale	Statistical model	Effect size	Quality score
Oddvar Solli 2010 [1]	Norway	Questionnaire survey	365	EQ-5D	Multiple logistic regression	OR and 95%CI	9
Jin Ook Chung 2013 [2]	Korea	Questionnaire survey	401	Audit of Diabetes Dependent Quality of Life (ADDQoL)	Multiple logistic regression	OR and 95%CI	7
Carlos K H Wong 2013 [5]	China	Questionnaire survey	1394	SF-12	Multiple linear regression	*β* and 95%CI	7
Aqil Mohammad Daher 2015 [11]	Malaysia	Questionnaire survey	256	Audit of Diabetes Dependent Quality of Life (ADDQoL)	Hierarchical multiple linear regression	*β* and 95%CI	7
Ji-Yan Cong 2012 [12]	China	Questionnaire survey	174	Diabetes Specific Quality of Life (DSQL)	Multiple logistic regression	OR and 95%CI	8
Aldona 2013 [13]	Lithuania	Questionnaire survey	1022	SF-36	Multiple logistic regression	OR and 95%CI	8
Pan 2013 [14]	China	Questionnaire survey	158	patients with Type 2 Diabetes Mellitus Quality of Life Scale (DMQLS)	Multiple logistic regression	OR and 95%CI	6
Anne Neumann 2014 [15]	Sweden	Questionnaire survey	43,586	SF-36	Beta regression	Coefficient and 95%CI	6
Maria Donald 2013 [16]	Australia	Questionnaire survey	3609	Audit of Diabetes Dependent Quality of Life (ADDQoL)	Multiple linear regression	*β* and 95%CI	7
Ayman 2014 [17]	Saudi Arabia	Questionnaire survey	283	SF-36	Multiple linear regression	*β* and 95%CI	6
Fuyong Hu 2015 [18]	China	Questionnaire survey	436	SF-36	Multiple logistic regression	OR and 95%CI	7
Liu 2009 [19]	China	Questionnaire survey	130	Diabetes Specific Quality of Life (DSQL)	Multiple logistic regression	OR and 95%CI	7
Zu 2015 [20]	China	Questionnaire survey	2741	SF-36	Multiple logistic regression	OR and 95%CI	7
Xie 2008 [21]	China	Questionnaire survey	217	Diabetes Specific Quality of Life (DSQL)	Multiple logistic regression	OR and 95%CI	6
Farzana Saleh 2015	Bangladesh	Questionnaire survey	500	EQ-5D	Multiple linear regression	*β* and 95%CI	6
W. Ken Redekop 2002 [22]	Netherlands	Questionnaire survey	1136	EQ-5D	Multiple logistic regression	OR and 95%CI	7
Nelda Mier 2008 [23]	United States of America	Questionnaire survey	399	SF-8	Multiple logistic regression	OR and 95%CI	9
Rebekah J. Walker 2015 [24]	United States of America	Questionnaire survey	302	SF-12 V	Multiple linear regression	*β* and 95%CI	6

More than one statistical methods could calculate the related factors of a dependent variable. In our selected studies, some studies used the multivariate logistic regression so their effect size was *OR* and 95%*CI*, and some used linear regression so their effect size was *β* and 95%*CI*.

In our 18 selected studies, there were 7 articles [2, 11, 14–18] reached the included criteria, but due to some reasons, their results could not be pooled by the method of meta-analysis, but they could be included into the systematic review. The reasons were as follows: One article [14] used a special scale (DMQLS, patients with Type 2 diabetes mellitus quality of life scale) to evaluate the QOL, which was different from all the other 17 studies. So its results could not be combined with the results of other studies with meta-analysis method. Three studies [15, 17, 18] used SF-36 scale and SF-12 scale, respectively. While their statistical methods were different from each other (Beta regression, multiple linear regression and multiple logistic regression, respectively). Three studies [2, 11, 16] used the same ADDQol (Audit of Diabetes Dependent Quality of Life) scale, while they used different statistical methods (Logistic regression, hierarchical multiple linear regression and multiple linear regression, respectively). So their effect sizes were different, and could not be combined together with meta-analysis method.

In addition, in the 11 articles of the meta-analysis [1, 5, 12, 13, 19–24], there were also many related factors did not be included to the meta-analysis because they were calculated in only one study, and some negative results were not exhibited in the articles.

Due to the reasons above. We did a systematic review together with meta-analysis to make the results more complete.

The summary of the results of meta-analysis and heterogeneity (*I*^*2*^) were showed in Table 2.

**Table 2:** Summary Results of Meta-analysis

Factors	Scales (Sub-scales)	Pooled ORs/ βs	95%CI	*I*^*2*^ (*%*)	Models
Complications	DSQL	3.038 ^a^	(1.956, 4.720)	0.0	Fixed Effects Model
	SF-36 (PF)	1.730 ^a^	(1.357, 2.204)	36.1	Fixed Effects Model
	SF-36 (RP)	1.516 ^a^	(1.199, 1.917)	0.0	Fixed Effects Model
	SF-36 (BP)	1.553 ^a^	(1.223, 1.973)	0.0	Fixed Effects Model
	SF-36 (GH)	1.704 ^a^	(1.335, 2.174)	0.0	Fixed Effects Model
	SF-36 (SF)	1.462 ^a^	(1.148, 1.862)	0.0	Fixed Effects Model
	SF-36 (RE)	2.611	(0.656, 10.387)	93.5	Random Effects Model
Hypertension	SF-36 (PF)	1.389 ^a^	(1.173, 1.644)	45.5	Fixed Effects Model
Duration of diabetes	SF-36 (PH)	1.865 ^a^	(1.088, 3.197)	0.0	Fixed Effects Model
Physical exercise	SF-36 (PF)	0.842	(0.319, 2.221)	95.6	Random Effects Model
	SF-36 (RP)	0.683 ^a^	(0.510, 0.913)	56.0	Random Effects Model
	SF-36 (BP)	0.662	(0.397, 1.104)	80.3	Random Effects Model
	SF-36 (GH)	0.660 ^a^	(0.567, 0.768)	0.0	Fixed Effects Model
	SF-36 (VT)	0.635 ^a^	(0.542, 0.745)	0.0	Fixed Effects Model
	SF-36 (SF)	0.825 ^a^	(0.711, 0.958)	0.0	Fixed Effects Model
	SF-36 (RE)	0.804	(0.463, 1.395)	86.7	Random Effects Model
	SF-36 (ES)	0.642 ^a^	(0.443, 0.929)	63.9	Random Effects Model
Diet with more red meat	DSQL	2.085 ^a^	(1.063, 4.089)	55.9	Random Effects Model
Diet control	SF-36 (PF)	1.064	(0.429, 2.643)	89.1	Random Effects Model
Glucose check frequently	SF-36 (PH)	0.175 ^a^	(0.041, 0.756)	0.0	Fixed Effects Model
Depression	DSQL	3.003 ^a^	(1.135, 7.948)	73.5	Random Effects Model
	SF-36 (PH)	5.667 ^a^	(3.184, 10.086)	0.0	Fixed Effects Model
	SF-36 (MH)	11.473 ^a^	(4.195, 31.383)	68.5	Random Effects Model

^a^: Statistically significant

### Characteristics related to the disease

#### Complications

**Fig. 2:**
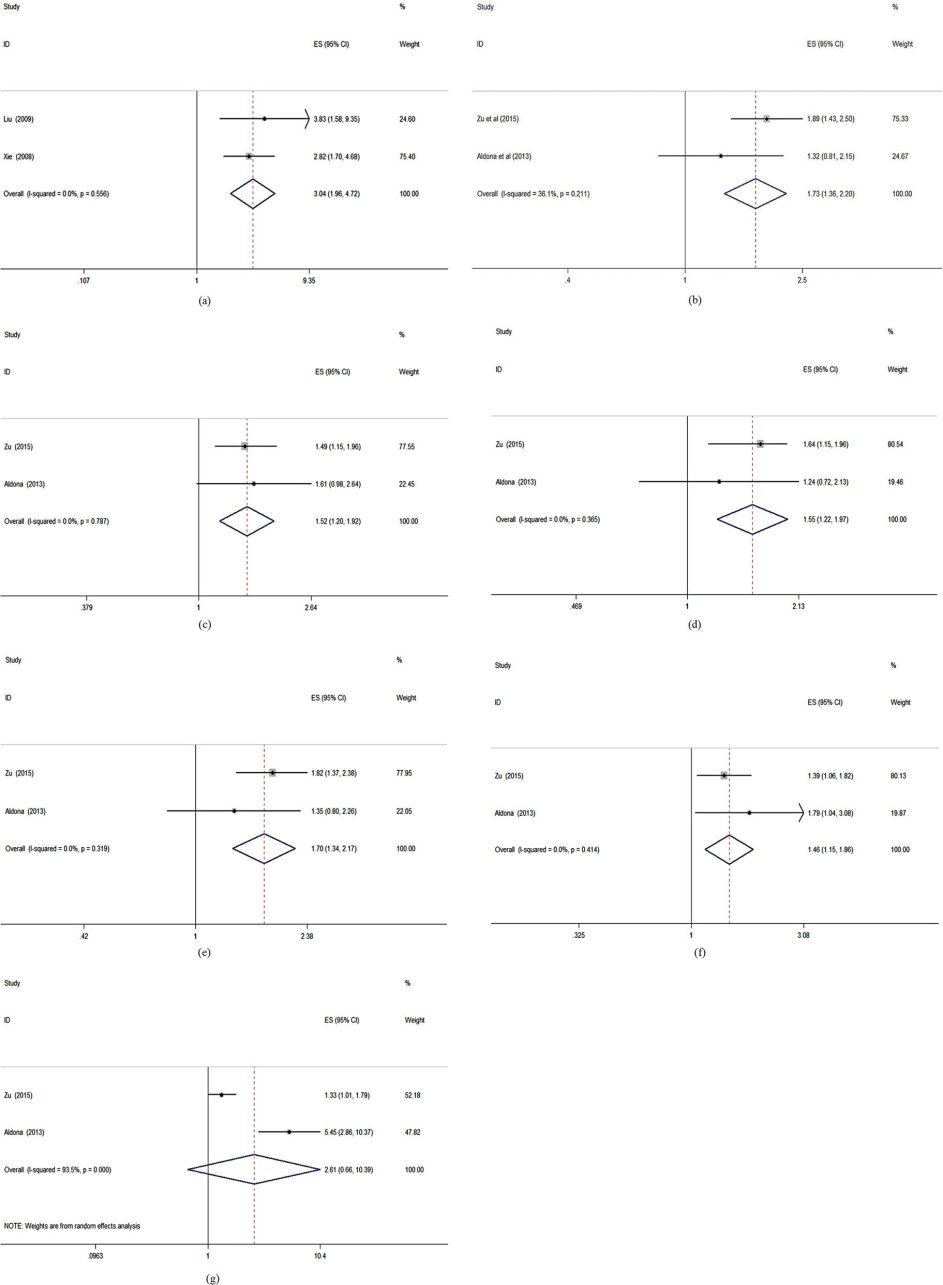
Forest plots of complications and the quality of life measured by different scales (ORs). Legends: (**a**) DSQL; (**b**) SF-36 (PF); (**c**) SF-36 (RP); (**d**) SF-36 (BP); (**e**) SF-36 (GH); (**f**) SF-36 (SF); (**g**) SF-36 (RE)

Figure 2 and Table 2 showed that compared with those type 2 diabetes patients without complication, those patients with complications had worse QOL measured by the DSQL scale (pooled OR = 3.038, 95%CI: 1.956–4.720) and on “Physical Functioning” (pooled OR = 1.730, 95%CI: 1.357–2.204) “Role limitations due to Physical health problems” (pooled OR = 1.516, 95%CI: 1.199–1.917) “Bodily Pain” (pooled OR = 1.553, 95%CI: 1.223–1.973) “General Health perceptions” (pooled OR = 1.704, 95%CI: 1.335–2.174) and “Social Functioning” (pooled OR = 1.462, 95%CI: 1.148–1.862) dimensions of SF-36.

Except the studies included the meta-analysis, other selected studies also reported the association between complications and QOL of type 2 diabetes patients. Five studies [1, 12, 17, 21, 22] showed that those patients with complications had worse QOL than those patients without complications. Especially, a significant gradient was reported for the number of complications and QOL in one study [16]. While there were also different opinions, one study [2] showed that microvascular complication has no association with QOL, and one study [11] showed that the relationships between QOL and renal complication and foot ulcer were not significant, respectively.

#### Hypertension

**Fig. 3:**
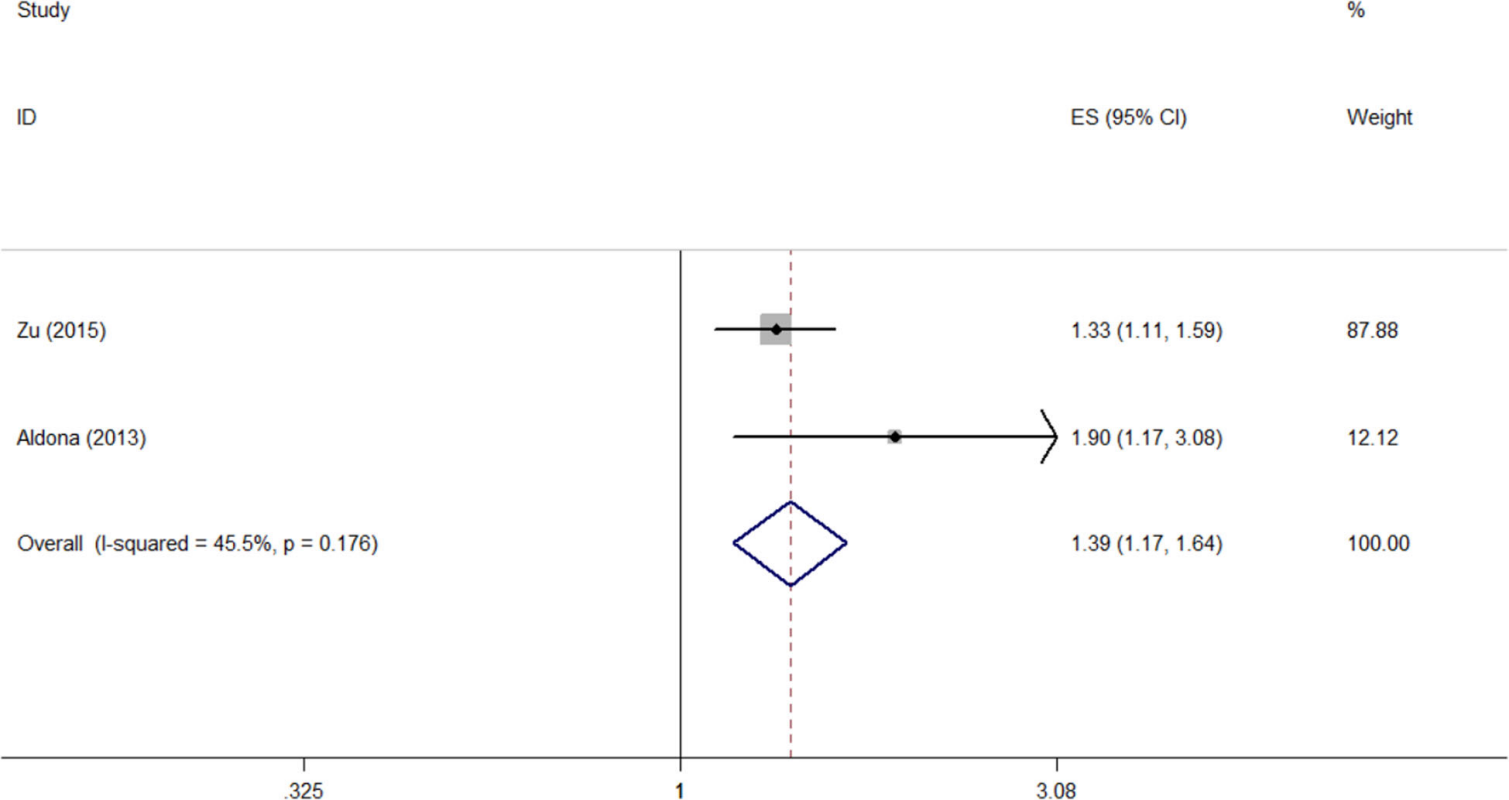
Forest plot of hypertension and the quality of life measured by SF-36 (PF) scale (OR)

Hypertension was directly associated in “Physical Functioning” of SF-36 scale (pooled OR = 1.389, 95%CI: 1.173–1.644). Compared with type 2 diabetes patients without hypertension, those with hypertension had worse QOL (Fig. 3 and Table 2).

Other selected studies also reported the relationship between hypertension and QOL of type 2 diabetes patients. One article [19] showed that hypertension was a risk factor of worse QOL. While two articles [11, 22] showed that their association was not statistically significant.

#### Duration of diabetes

**Fig. 4:**
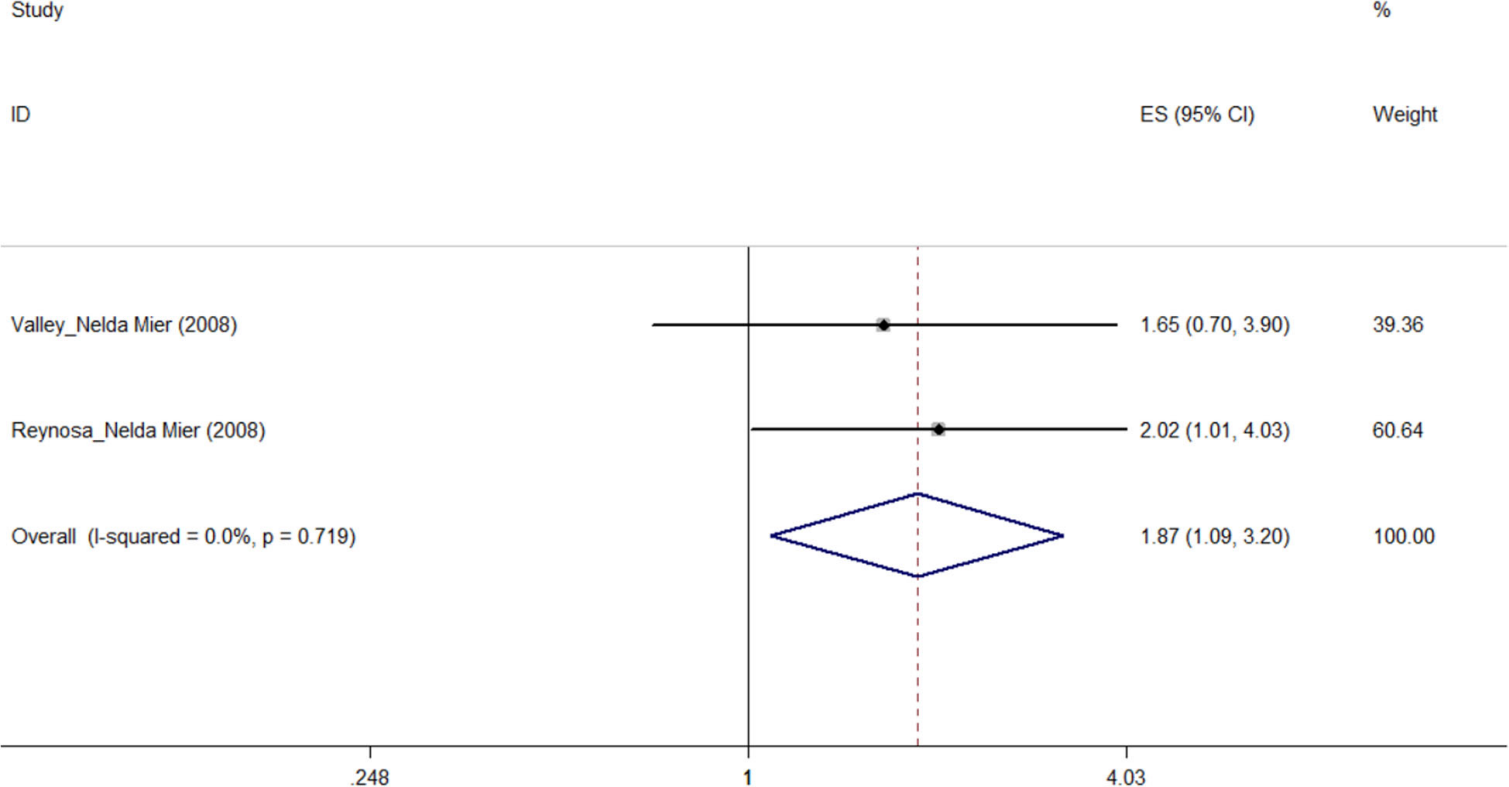
Forest plot of duration and the quality of life measured by SF-36 (PH) scale (OR)

According to Fig. 4 and Table 2, for QOL measured by the “Physical Health” sub-scale of SF-36 scale, the longer duration of diabetes was associated with worse QOL. “Had duration more than 10 years” was a predictor of worse physical health status than those whose duration were less than 10 years (pooled OR = 1.865, 95%CI: 1.088–3.197).

The conclusions that if the duration was a risk factor of QOL or not were different. In our selected studies, four studies [17, 22, 24] reported the relationship between them was not statistically significant. While two studies [11, 13] showed that long duration, especially more than 10 year duration, was associated with worse QOL of type 2 diabetes patients significantly.

#### Insulin use

According to the selected articles which mentioned the relationship between insulin use and QOL of type 2 diabetes patients, three of them [11, 13, 22] showed that patients who used insulin had a worse QOL than those patient did not use insulin. While one article [2] reported that the relationship between them was not significantly.

### Life styles

#### Physical exercise

**Fig. 5:**
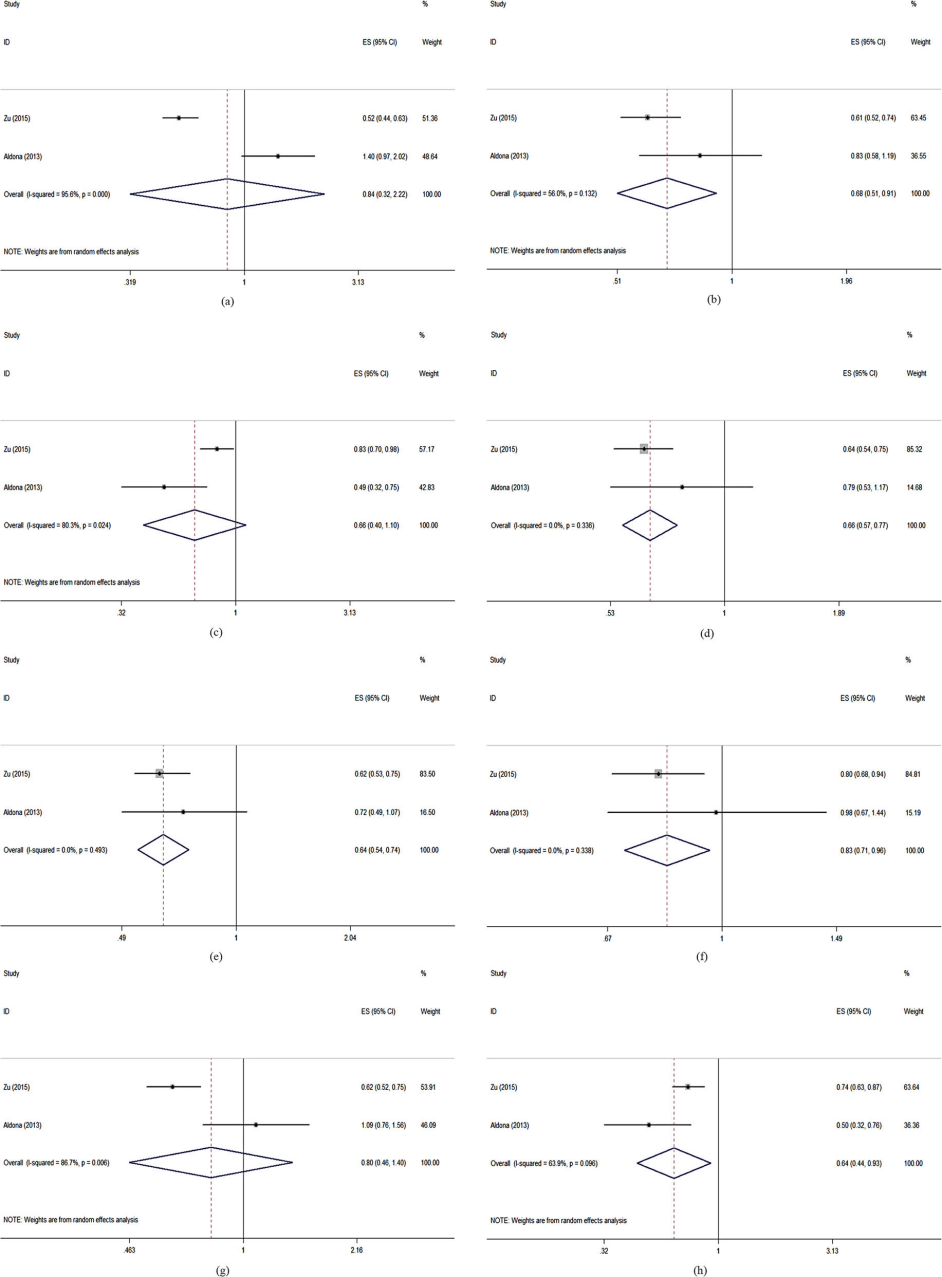
Forest plots of physical exercise and the quality of life measured by different scales (ORs). Legends: (**a**) SF-36 (PF); (**b**) SF-36 (RP); (**c**) SF-36 (BP); (**d**) SF-36 (GH); (**e**) SF-36 (VT); (**f**) SF-36 (SF); (**g**) SF-36 (RE); (**h**) SF-36 (ES)

After our calculation, patients who did more physical exercise had a better QOL than those who did less physical exercise in many sub-scales measured by SF-36 scale as follows: “Role limitations due to Physical health problems” (pooled OR = 0.683, 95%CI: 0.510–0.913), “General Health perceptions” (pooled OR = 0.660, 95%CI: 0.567–0.768), “Vitality” (pooled OR = 0.635, 95%CI: 0.542–0.745), “Social Functioning” (pooled OR = 0.825, 95%CI: 0.711–0.958), and “Emotional State” (pooled OR = 0.642, 95%CI: 0.443–0.929). While it did not have effects on another three sub-scales (Fig. 5 and Table 2).

#### Diet with more red meat

**Fig. 6:**
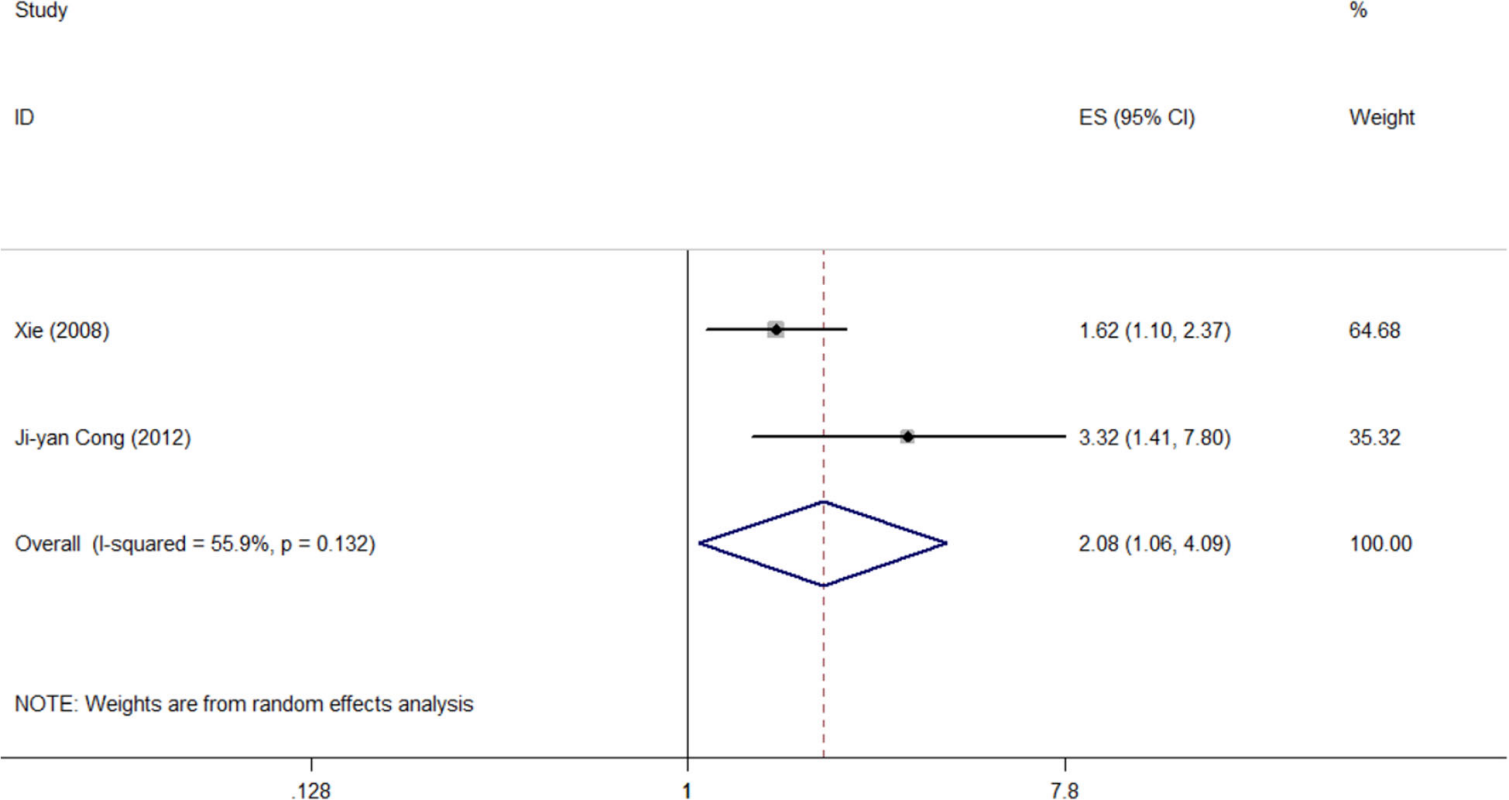
Forest plot of diet with more red meat and the quality of life measured DSQL scale (OR)

Measured by the DSQL scale, “Diet with more red meat” was a negative factor on QOL according to our calculation. As is shown in Fig. 6 and Table 2, the pooled OR was 2.085, and the 95%CI was (1.063, 4.089).

#### Diet control

**Fig. 7:**
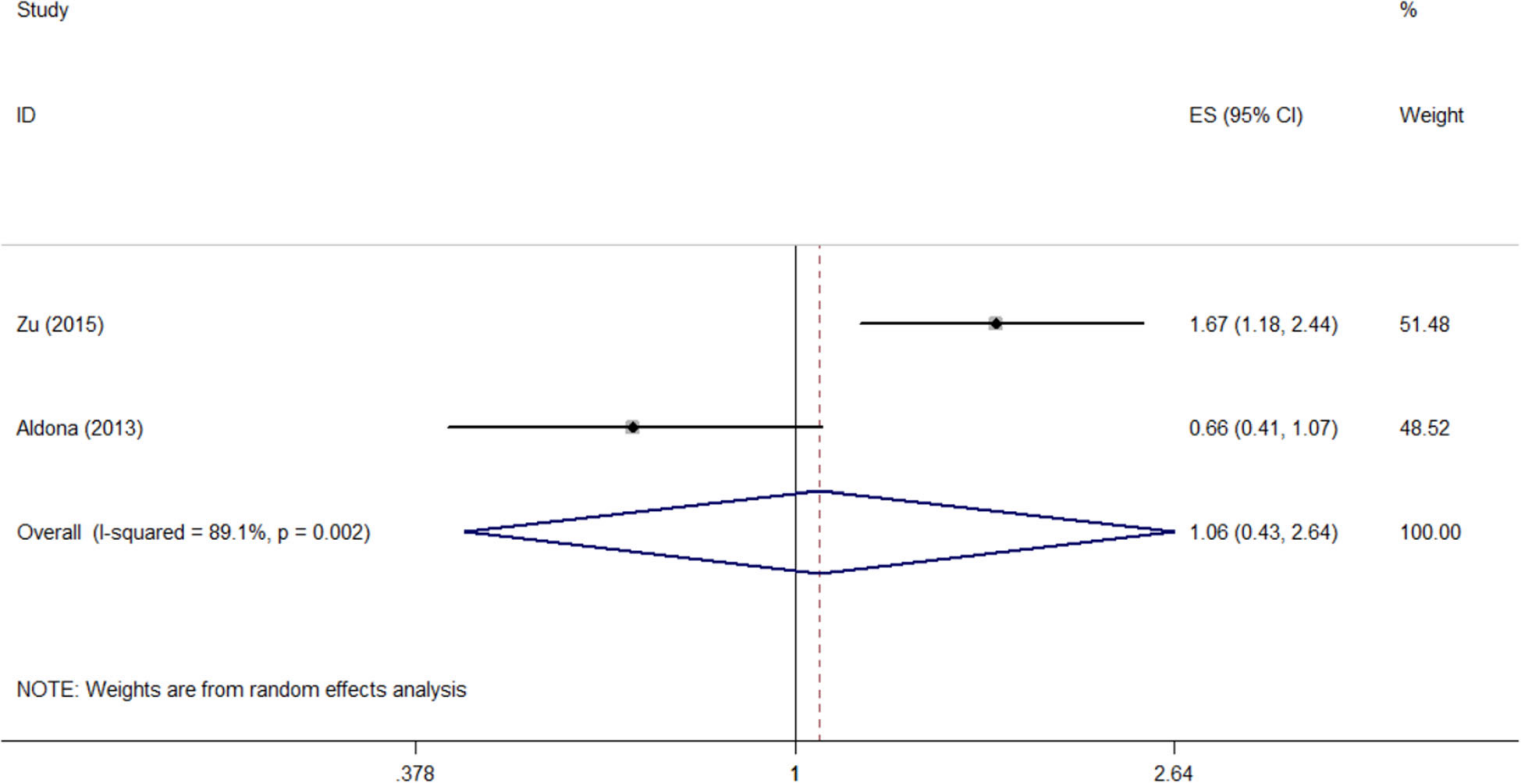
Forest plot of diet control and the quality of life measured by SF-36 (PF) scale (OR)

After our calculation, “Diet control” did not associated with QOL on “Physical Functioning” measured by the SF-36 scale (pooled OR = 1.064, 95%CI: 0.429–2.643). The OR value showed that “Diet control” could bring worse QOL, while its 95%CI was not significant (Fig. 7 and Table 2).

#### Glucose check frequently

**Fig. 8:**
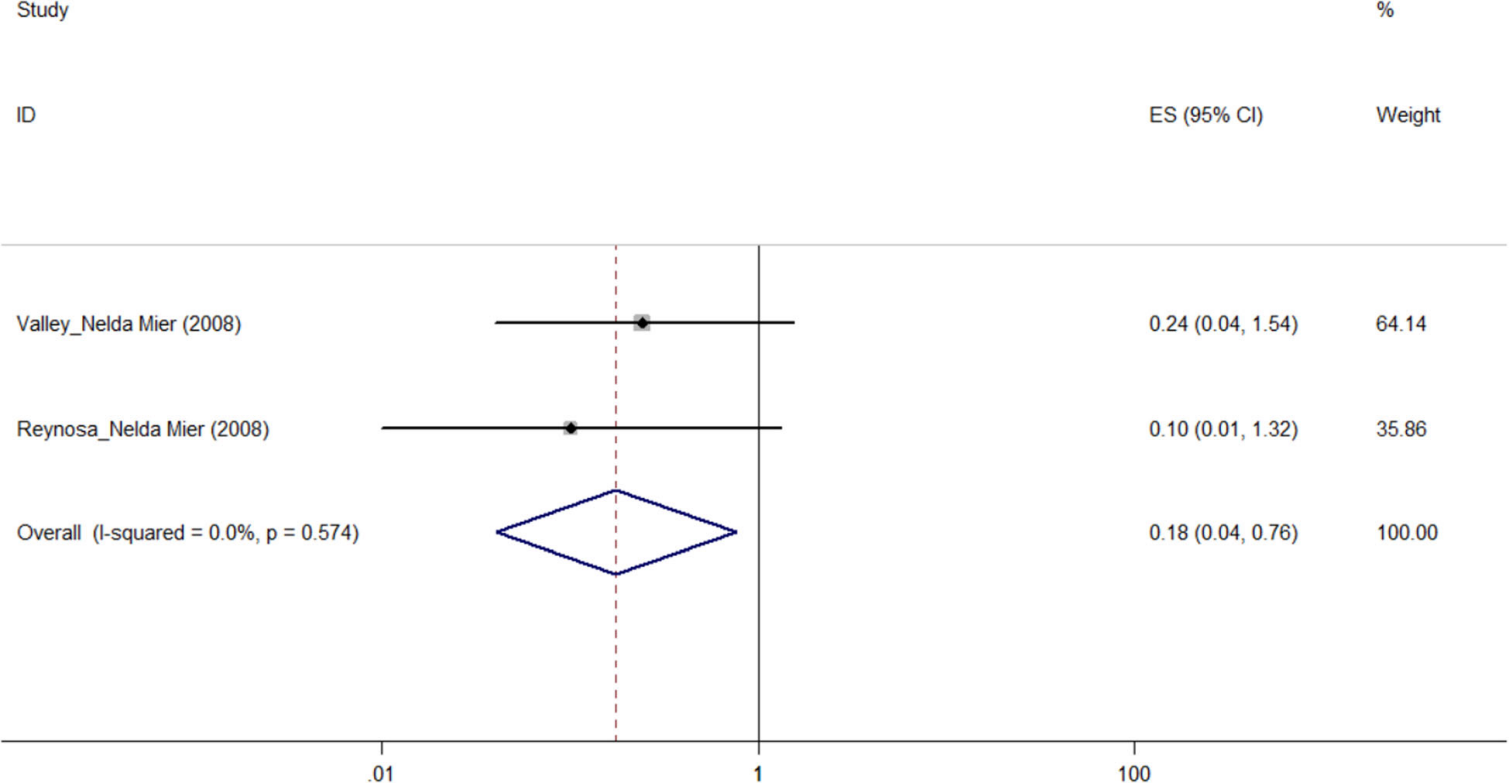
Forest plot of glucose check frequently and the quality of life measured by SF-36 (PH) scale (OR)

According to Fig. 8 and Table 2, although the included studies showed that “Glucose check frequently” did not associated with QOL measured by the “Physical Health” sub-scale of SF-36 scale, our pooled result indicated that patients who checked their glucose frequently had a better QOL than those did not(pooled OR = 0.175, 95%CI: 0.041–0.756).

### Mental factors

#### Depression

**Fig. 9:**
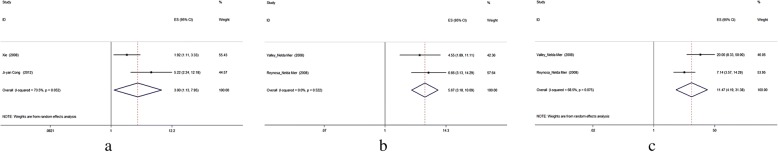
Forest plot of depression and the quality of life measured by different scales (ORs). Legends: (**a**) DSQL; (**b**) SF-36 (PH); (**c**) SF-36 (MH)

Depression was directly associated with QOL measured by the DSQL scale (pooled OR = 3.003, 95%CI: 1.135–7.948), by the “Physical Health” (pooled OR = 5.667, 95%CI: 3.184–10.086) and “Mental Health” (pooled OR = 11.473, 95%CI: 4.195–31.383) sub-scales of SF-36 scale (Fig. 9 and Table 2).

Same to the result of meta-analysis, after our systematic review, there were also three studies [14, 16, 24] showed that depression would cause worse QOL of type 2 diabetes patients.

#### Anxiety and worry

Similar to depression, our selected article [14, 16] showed that anxiety was a risk factor of worse QOL of type 2 diabetes patients. Also, one article [11] reported that being worried about the disease could cause worse QOL.

##### Publication bias results

Egger’s test was used to evaluate the publication bias in our selected studies. Our study included many scales and many influencing factors of QOL. So we calculated the publication bias according to the scales and factors, respectively. The results of Egger’s test were shown in Table 3. It did not show evidence of publication bias in our study.

**Table 3:** Publication bias test results for included studies of meta-analysis

Studies	Egger’s Statistics	Publication bias
Liu 2009 & Xie 2008	1.567	None
Zu 2015 & Aldona 2013	−3.380	None
W. Ken. Redekop 2002 & Oddvar Solli 2010	0.951	None
Farzana Saleh 2015 & Oddvar Solli 2010	−0.245	None
Xie 2008 & Ji-yan Cong 2012	2.985	None
Rebekah J. Walker 2015 & Carlos K H Wong 2013	−3.970	None

## Discussion

Type 2 diabetes patients were expected to improve their QOL via self-management and life-time metabolic control [10]. The QOL was gaining importance as the physiological or clinical outcome parameter [10]. Therefore, one of the objectives in the management of diabetes was to minimize the deterioration in the QOL [2]. The aim of our study was to find out the related factors of QOL of type 2 diabetes patients (including characteristics related to the disease, life styles and mental health factors).

After our search, 18 articles were entered into our systematic review and meta-analysis. The 18 studies contained 11 countries and 57,109 research objects, using 5 kinds of scales. Opinions in these studies were not totally same to each other.

After the analysis, we found that complications could affect the QOL of type 2 diabetes patients at almost all aspects. Tang et al. [33], Shiu et al. [34], and Wexler et al. [35] also showed the result that the QOL of T2DM was lower if the patients showed complications. Complications could affect the QOL of type 2 diabetes patients in many ways, such as increasing physical discomfort, decreasing their activity, and reducing their physical state. In addition, these various complications could extend treatment time and add therapy methods [12]. For instance, the treatment of type 2 diabetes with end-stage nephropathy required not only medical therapy, but also dialysis and even renal transplant [36]. Meanwhile, complications could increase the cost of type 2 diabetes [36–38]. Therefore, complications may increase the material and mental burden of type 2 diabetes patients. Besides, depression also could cause more complications [38]. So depression may cause worse QOL of type 2 diabetes patients. It was similar to the result of our study, depression was associated with lower QOL score. The results of studies of Wexler et al. [35] and Verma et al. [39] also showed depression was an associated characteristic of QOL of T2DM. Overall, complications and depression could affect the QOL of type 2 diabetes patients together. It was of interest that whether depression should be considered as complication of diabetes rather than comorbidity [16]. Similarly, for type 2 diabetes patients, knowing adequate information about the natural history of the disease was helpful to develop a positive attitude toward type 2 diabetes [11]. Fear of hypoglycemia could influence the patients to maintain a high blood glucose level [40–42]. So worry about the disease could be seen as one of the factors that may cause worse QOL.

The relationship between duration of diabetes and the QOL was still controversial [17]. According to our study, the longer duration could cause the worse QOL. Some studies also reported that increased duration of diabetes was associated with poor QOL in T2DM patients [43]. It may be caused by that glycaemia control tended to be worse with longer duration due to a decline in beta cell function, and a decline in patients’ attitude and adherence to treatment regimen [11]. Some previous studies [44–46] reported that glycaemia control was an important determinant of QOL. While checking glucose frequently could be helpful for glycaemia control, so glucose check frequently might be a preventive factor for QOL [11], similar to our study.

According to our study, physical exercise was preventive to the QOL on most dimensions of SF-36 scale. Physical exercise is beneficial for health in any domain (recreation, transportation and so on) and is recommended by the WHO [47]. It could help to reduce the risk of diabetes [48], and is correlated with blood glucose and blood pressure control [12, 49]. Sung et al. [50] found that a regular walking was effective for lowering blood glucose and HbA1c in elderly people with type 2 diabetes.

According to our study, diet control had no significant association with the QOL of type 2 diabetes patients. While diet with more red meat was a negative factor of the QOL. A previous study reported that people in the top quintile of red meat intake had a greater chance of having a metabolic syndrome [51]. The mechanism of the relationship between eating more red meat and QOL was not clearly understood. It was possible a surrogate of some other influenced factors [12]. For example, the meat-eater could had higher BMI than other people [12], and women with high intake of red meat tended to have less likely to exercise [52].

The strongest strength of our study was that it was the first meta-analysis about related factors of the QOL of type 2 diabetes patients. We calculated as many factors as possible, and we classified these factors into 3 groups to make it much clear to understand. Some variables had wide range confidence intervals [such as complications measured by SF-36-Role limitations due to Emotional problems (0.656, 10.387), Depression measured by SF-36-Physical Health (3.184, 10.086) and Mental Health (4.195, 31.383)]. It was because the number of the included researches for each factor was too small. And in meta-analysis, the results with wide confidence interval were always treated as moderate-quality evidences [53, 54]. So it need more related studies to find out the real association between these factors and QOL of type 2 diabetes patients.

Meanwhile, our study had several limitations. First, due to the language restriction, we included the publications in English and Chinese only. So articles written in other languages were ignored. Second, the included studies have used many different QOL scales and the objective factors of each study was not exactly identical, so many results in our study were pooled by only 2 included articles. Third, in many our included studies, the authors have not showed the negative results. They only reported the factors associated with the QOL significantly. So much information was missing. Fourth, heterogeneity among these included studies may affect the accuracy of our results. Sensitivity analysis and meta-regression could help to find the source of heterogeneity. However, limited to the dispersion and the number of studies, we could not calculate it exactly.

## Conclusion

We analyzed the related factors of QOL of type 2 diabetes patients using 18 included studies via systematic review and meta-analysis, and found some interesting results. We classified these factors into 3 groups (characteristics related to the disease, life styles and mental factors). The mechanism of the relationships between these factors and QOL was complicated. Making targeted strategies on these factors could improve the QOL of type 2 diabetes patients more efficiently. And we hope that more researchers could focus the QOL of type 2 diabetes patients, meanwhile, an authoritative guideline for improving the QOL of type 2 diabetes patients could be issued soon in the future.

## Abbreviations

95% CI: 95% confidence intervals
ADDQoL: Audit of Diabetes Depentent Quality of Life
BMI: Body mass index
BP: Bodily Pain
CNKI: China National Knowledge Infrastructure
DSQL: Diabetes-specific quality of life
EQ-5D: EuroQol 5D
ES: Emotional State
GH: General Health perceptions
MH: Mental Health
PF: Physical Functioning
PH: Physical Health
QOL: Quality Of Life
RE: Role limitations due to Emotional problems
RP: Role limitations due to Physical health problems
SF: Social Functioning
SF-8 SF-12 SF-36: Short Form-8 Short Form-12 Short Form-36
VT: Vitality
WHO: World Health Organization

## References

[1] Solli O, Stavem K, Kristiansen IS (2010). Health-related quality of life in diabetes: the associations of complications with EQ-5D scores. Health Qual Life Outcomes.

[2] Chung JO, Cho DH, Chung DJ, Chung MY (2013). Assessment of factors associated with the quality of life in Korean type 2 diabetic patients. Intern Med.

[3] Wild S, Roglic G, Green A, Sicree R, King H (2004). Global prevalence of diabetes: estimates for the year 2000 and projections for 2030. Diabetes Care.

[4] World Health Organization. 10 facts on diabetes [EB/OL]. Available at: http://www.who.int/features/factfiles/diabetes/en/ April 2016.

[5] Wong CK, Lo YY, Wong WH, Fung CS (2013). The associations of body mass index with physical and mental aspects of health-related quality of life in Chinese patients with type 2 diabetes mellitus: results from a cross-sectional survey. Health Qual Life Outcomes.

[6] Dickerson F, Wohlheiter K, Medoff D (2011). Predictors of quality of life in type 2 diabetes patients with schizophrenia, major mood disorder, and without mental illness. Qual Life Res.

[7] Rubin R, Peyrot M (1999). Quality of life and diabetes. Diab Metabol Res Rev.

[8] Schram MT, Baan CA, Pouwer F (2009). Depression and quality of life in patients with diabetes: a systematic review from the European depression in diabetes (EDID) research consortium. Curr Diabetes Rev.

[9] Polonsky W (2002). Emotional and quality-of-life aspects of diabetes management. Curr Diab Rep.

[10] Saleh F, Ara F, Mumu SJ, Hafez MA (2015). Assessment of health-related quality of life of Bangladeshi patients with type 2 diabetes using the EQ-5D: a cross-sectional study. BMC Res Notes.

[11] Daher AM, AlMashoor SA, Winn T (2015). Glycaemic control and quality of life among ethnically diverse Malaysian diabetic patients. Qual Life Res.

[12] Cong JY, Zhao Y, Xu QY, Zhong CD, Xing QL (2012). Health-related quality of life among Tianjin Chinese patients with type 2 diabetes: a cross-sectional survey. Nurs Health Sci.

[13] Mikailiukstiene A, Juozulynas A, Narkauskaite L, Zagminas K, Salyga J, Stukas R (2013). Quality of life in relation to social and disease factors in patients with type 2 diabetes in Lithuania. Med Sci Monit.

[14] Pan X, Hong Z, Xu R (2013). Relationship between negative emotion and quality of life of type 2 diabetes patients. J Qilu Nur.

[15] Neumann A, Schoffer O, Norstrom F, Norberg M, Klug SJ, Lindholm L (2014). Health-related quality of life for pre-diabetic states and type 2 diabetes mellitus: a cross-sectional study in Vasterbotten Sweden. Health Qual Life Outcomes.

[16] Donald M, Dower J, Coll JR, Baker P, Mukandi B, Doi SA (2013). Mental health issues decrease diabetes-specific quality of life independent of glycaemic control and complications: findings from Australia's living with diabetes cohort study. Health Qual Life Outcomes.

[17] Al Hayek AA, Robert AA, Al SA, Alzaid AA, Al Sabaan FS (2014). Factors associated with health-related quality of life among Saudi patients with type 2 diabetes mellitus: a cross-sectional survey. Diabetes Metab J.

[18] Hu F, Niu L, Chen R, Ma Y, Qin X, Hu Z (2015). The association between social capital and quality of life among type 2 diabetes patients in Anhui province, China: a cross-sectional study. BMC Public Health.

[19] Yiming L (2009). Survey and analysis on factors affecting living quality of type 2 diabetes. Anhui Med J.

[20] Ping Z, Linag S, Yan S, Weijian Z (2015). Quality of life and influencing factors in patients with type 2 diabetes in communities of Shanghai. J Environ Occu Med.

[21] Xie Y, Wang J (2008). Study on quality of life and influencing factors in patients with type 2 diabetes mellitus in community. Chin J Dis Control Prev.

[22] Redekop WK, Koopmanschap MA, Stolk RP, Rutten GE, Wolffenbuttel BH, Niessen LW (2002). Health-related quality of life and treatment satisfaction in Dutch patients with type 2 diabetes. Diabetes Care.

[23] Mier N, Bocanegra-Alonso A, Zhan D, Zuniga MA, Acosta RI (2008). Health-related quality of life in a binational population with diabetes at the Texas-Mexico border. Rev Panam Salud Publica.

[24] Walker RJ, Lynch CP, Strom Williams J, Voronca D, Egede LE (2015). Meaning of illness and quality of life in patients with type 2 diabetes. J Diabetes Complicat.

[25] World Health Organization (1999). Definition, diagnosis and classification of diabetes mellitus and its complications: report of a WHO consultation. Part 1: diagnosis and classification of diabetes mellitus.

[26] Hu J, Dong Y, Chen X, Liu Y, Ma D, Liu X, Zheng R, Mao X, Chen T, He W (2015). Prevalence of suicide attempts among Chinese adolescents: a meta-analysis of cross-sectional studies. Compr Psychiatry.

[27] Aaronson NK, Muller M, Cohen PD (1998). Translation, validation, and norming of the Dutch language version of the SF-36 health survey in community and chronic disease populations. J Clin Epidemiol.

[28] Cohen JA, Beall D, Beck A (1999). Sumatriptan treatment for migraine in a health maintenance organization: economic, humanistic, and clinical outcomes. Clin Ther.

[29] Dahlof C, Bouchard J, Cortelli P (1997). A multinational investigation of the impact of subcutaneous sumatriptan. II: health-related quality of life. Pharmaco Econ.

[30] Lang E, Kastner S, Neundorfer B, Bickel A (2001). Konnen Therapieempfehlungen oder Patientenseminare die Effektivitat der ambulanten Versorgung von Patienten mit Kopfschmerzen verbessern? Effects of recommendations and patient seminars on effectivity of outpatient treatment for headache. Schmerz.

[31] Lofland JH, Johnson NE, Batenhorst AS, Nash DB (1999). Changes in resource use and outcomes for patients with migraine treated with sumatriptan: a managed care perspective. Arch Intern Med.

[32] Turner-Bowker DM, Bayliss MS, Ware JE, Kosinski M (2003). Usefulness of the SF-8 health survey for comparing the impact of migraine and other conditions. Qual Life Res.

[33] Tang WL, Wang YM, Du WM, Cheng NN, Chen BY (2006). Assessment of quality of life and relevant factors in elderly diabetic patients in the Shanghai community. Pharmacoepidemiol Drug Saf.

[34] Shiu AT, Thompson DR, Wong RY (2008). Quality of life and its predictors among Hong Kong Chinese patients with diabetes. J Clin Nurs.

[35] Wexler DJ, Grant RW, Wittenberg E (2006). Correlates of healthrelated quality of life in type 2 diabetes. Diabetologia.

[36] Brandle M, Zhou H, Smith BR (2003). The direct medical cost of type 2 diabetes. Diabetes Care.

[37] Happich M, John J, Stamenitis S (2008). The quality of life and economic burden of neuropathy in diabetic patients in Germany in 2002 – results from the diabetic microvascular complications (DIMICO) study. Diabetes Res Clin Pract.

[38] de Groot M, Anderson R, Freedland KE, Clouse RE, Lustman PJ (2001). Association of depression and diabetes complications: a metaanalysis. Psychosom Med.

[39] Verma SK, Luo N, Subramaniam M (2010). Impact of depression on health related quality of life in patients with diabetes. Ann Acad Med Singap.

[40] Adriaanse MC, Dekker JM, Spijkerman AM (2005). Diabetesrelated symptoms and negative mood in participants of a targeted population-screening program for type 2 diabetes: the hoorn screening study. Qual Life Res.

[41] Cryer PE (2008). The barrier of hypoglycemia in diabetes. Diabetes.

[42] Lundkvist J, Berne C, Bolinder B, Jönsson L (2005). The economic and quality of life impact of hypoglycemia. Eur J Health Econ.

[43] Glasgow RE, Ruggiero L, Eakin EG, Dryfoos J, Chobanian L (1997). Quality of life and associated characteristics in a large national sample of adults with diabetes. Diabetes Care.

[44] Shim YT, Lee J, Toh MPHS, Tang WE, Ko Y (2012). Health-related quality of life and glycaemic control in patients with type 2 diabetes mellitus in Singapore. Diabetic Med.

[45] Weinberger M, Kirkman MS, Samsa GP, Cowper PA, Shortliffe EA, Simel DL, Feussner JR (1994). The relationship between glycemic control and health related quality of life in patients with non-insulin-dependent diabetes mellitus. Med Care.

[46] U.K. Prospective diabetes study group (1999). Quality of life in type 2 diabetic patients is affected by complications but not by intensive policies to improve blood glucose or blood pressure control (UKPDS 37). Diabetes Care.

[47] World Health Organization. Global recommendations on physical activity for health. WHO, 2017.02. http://www.who.int/dietphysicalactivity/publications/9789241599979/en/26180873

[48] Kyu HH, Bachman VF, Alexander LT (2016). Physical activity and risk of breast cancer, colon cancer, diabetes, ischemic heart disease, and ischemic stroke events: systematic review and dose-response meta-analysis for the global burden of disease study 2013. BMJ.

[49] Colberg SR, Sigal RJ, Fernhall B (2010). Exercise and type 2 diabetes: the American College of Sports Medicine and the American Diabetes Association: joint position statement. Diabetes Care.

[50] Sung Kiwol, Bae Sangkeun (2012). Effects of a regular walking exercise program on behavioral and biochemical aspects in elderly people with type II diabetes. Nursing & Health Sciences.

[51] Azadbakht L, Esmaillzadeh A (2009). Red meat intake is associated with metabolic syndrome and the plasma C-reactive protein concentration in women. J Nutr.

[52] Song Y, Manson JE, Buring JE, Liu S (2004). A prospective study of red meat consumption and type 2 diabetes in middle-aged and elderly women: the women’s health study. Diabetes Care.

[53] Blanco GF, Delgado SG, Mongua RN, Cruz HP, Ferreyra RL, Ferreira GE, Yanes LM, Montero CR, Bobadilla DM, Torres GP, Ponce DA, Sifuentes OJ, Garcia GL (2017). Molecular clustering of patients with diabetes and pulmonary tuberculosis: a systematic review and meta-analysis. PLoS One.

[54] Howcroft M, Walters EH, Wood-Baker R, Walters JA (2016). Action plans with brief patient education for exacerbations in chronic obstructive pulmonary disease. Cochrane Database Syst Rev.

